# Early deafness leads to re-shaping of functional connectivity beyond the auditory cortex

**DOI:** 10.1007/s11682-020-00346-y

**Published:** 2020-07-23

**Authors:** Kamil Bonna, Karolina Finc, Maria Zimmermann, Lukasz Bola, Piotr Mostowski, Maciej Szul, Pawel Rutkowski, Wlodzislaw Duch, Artur Marchewka, Katarzyna Jednoróg, Marcin Szwed

**Affiliations:** 1 Centre for Modern Interdisciplinary Technologies, Nicolaus Copernicus University, 87-100 Toruń, Poland; 2 Faculty of Physics, Astronomy and Informatics, Nicolaus Copernicus University, 87-100 Toruń, Poland; 3 Department of Psychology, Jagiellonian University, 30-060 Krakow, Poland; 4 Section for Sign Linguistics, Faculty of Polish Studies, University of Warsaw, 00-927 Warsaw, Poland; 5 Laboratory of Brain Imaging, Neurobiology Center, Nencki Institute of Experimental Biology, Polish Academy of Sciences, 02-093 Warsaw, Poland; 6 Laboratory of Psychophysiology, Neurobiology Center, Nencki Institute of Experimental Biology, Polish Academy of Sciences, 02-093 Warsaw, Poland

**Keywords:** Brain plasticity, Deafness, Functional connectivity, Graph theory, Resting-state fMRI

## Abstract

Early sensory deprivation, such as deafness, shapes brain development in multiple ways. Deprived auditory areas become engaged in the processing of stimuli from the remaining modalities and in high-level cognitive tasks. Yet, structural and functional changes were also observed in non-deprived brain areas, which may suggest the whole-brain network changes in deaf individuals. To explore this possibility, we compared the resting-state functional network organization of the brain in early deaf adults and hearing controls and examined global network segregation and integration. Relative to hearing controls, deaf adults exhibited decreased network segregation and an altered modular structure. In the deaf, regions of the salience network were coupled with the fronto-parietal network, while in the hearing controls, they were coupled with other large-scale networks. Deaf adults showed weaker connections between auditory and somatomotor regions, stronger coupling between the fronto-parietal network and several other large-scale networks (visual, memory, cingulo-opercular and somatomotor), and an enlargement of the default mode network. Our findings suggest that brain plasticity in deaf adults is not limited to changes in the auditory cortex but additionally alters the coupling between other large-scale networks and the development of functional brain modules. These widespread functional connectivity changes may provide a mechanism for the superior behavioral performance of the deaf in visual and attentional tasks.

**Electronic supplementary material:**

The online version of this article (10.1007/s11682-020-00346-y) contains supplementary material, which is available to authorized users.

## Introduction

The lack of input from one sensory modality profoundly impacts brain development (Bavelier and Neville 2002; Merabet and Pascual-Leone 2010). In the case of deafness, the auditory cortex becomes involved in the processing of stimuli from remaining modalities, such as tactile or visual (Auer Jr et al. 2007; Bola et al. 2017; Finney et al. 2001; Karns et al. 2012; Levänen et al. 1998; Petitto et al. 2000). Auditory deprived areas also become engaged in higher-level cognitive tasks such as sign language processing (Nishimura et al. 1999; Trumpp and Kiefer 2018), speechreading (Capek et al. 2008; MacSweeney et al. 2001), visual attention (Bavelier et al. 2000), and working memory (Ding et al. 2015). This functional reorganization is accompanied by anatomical changes in sensory-deprived primary and secondary auditory areas (Emmorey et al. 2003; Finkl et al. 2020).

Alterations in the brain structure and function of deaf individuals are not restricted to the auditory system. In terms of brain structure, the deaf also displays an increased volume of the frontal areas (Leporé et al. 2010), the insula (Allen et al. 2008) and decreased gray matter volume in the occipital cortex (Pénicaud et al. 2013). Deaf signers, compared to hearing controls, have weaker structural connectivity in sensory-motor areas involved in the perception and production of speech (Finkl et al. 2020). In terms of brain function, deaf individuals display increased recruitment of multimodal parietal and occipital areas during performance of attention tasks (Bavelier et al. 2000, 2001; Neville and Lawson 1987b), and increased recruitment of the insula, anterior cingulate and thalamus during verbal memory tasks (Bavelier et al., 2008a, b). As the presented evidence suggests, changes caused by auditory input deprivation extend beyond auditory cortices, affecting non-deprived brain areas. Yet, the possible impact of these changes on whole-brain network architecture has not received adequate attention.

Resting-state fMRI (rsfMRI) offers a solution to investigate the whole-brain functional network organization with no explicit task requirements (van den Heuvel and Hulshoff Pol 2010). Using rsfMRI data, one can estimate functional connectivity (FC) between different brain areas by measuring the temporal dependence of the low-frequency (< 0.1 Hz) MRI signal fluctuations among them (Biswal et al. 1995). A recent study on deaf individuals found an increased resting-state FC between the right auditory cortex (superior temporal gyrus, STG) and key nodes of the salience network: the anterior insula and the dorsal anterior cingulate cortex (dACC) (Ding et al. 2016). Altered functional connectivity between the STG and the fronto-parietal network (FPN), that consists of nodes in lateral prefrontal and posterior parietal cortices, was also found in a task-based and resting-state study by Cardin et al. (2018). Other researchers reported altered functional connectivity in the deaf between medial temporal gyri and areas of the default mode network (DMN), with its key nodes in the posterior cingulate cortex (PCC) and the medial prefrontal cortex (Malaia et al. 2014). Increased resting-state functional connections were reported between the right superior parietal gyrus (rSPG) and the right insula, and between the middle temporal gyrus and the posterior cingulate gyrus (2016). In the same study, Li et al. (2016) also reported an altered arrangement of highly interconnected functional hubs. Specifically, in deaf adolescents, hubs were located in the superior and middle frontal gyri and cuneus, as opposed to precentral gyrus, hippocampus, and supramarginal gyrus in the hearing control group. Altered resting-state functional connections were also observed across the entire cortex of deaf cats, including not only auditory, but also visual, cingulate, and somatosensory networks (Stolzberg et al. 2018).

Collectively, these studies suggest that changes in the resting-state functional connectivity of the deaf may extend to other large-scale networks, particularly salience, FPN, and DMN. These three networks are suggested to cooperate during demanding cognitive tasks that require cognitive control: salience network is responsible for dynamical switching between the FPN (task-positive network) and the DMN (task-negative network) (Sridharan et al. 2008). As deaf individuals display superior performance in attention (Bavelier et al. 2000) and visuospatial working memory (Ding et al. 2015), we may expect altered connectivity patterns between these three networks.

Network neuroscience studies revealed that the brain network is organized in a modular way, where highly interlinked regions with similar functions form large-scale networks (Sporns 2013). The modular organization of the brain network promotes efficient information processing and adaptability in a changing environment (Sporns and Betzel 2016). Functional brain modules are shaped during neurodevelopment in a way that within-module connections become stronger, while between-module connections become weaker (Baum et al. 2017). We might expect that the functional brain network’s level of segregation may be shaped by the kind of sensory information received by the system during development. The consequence of early sensory deprivation on the development of functional brain modules, is still unknown.

The goal of the present study was to examine differences between the whole-brain functional networks of early deaf and hearing adults. First, we were interested in whether early deafness may alter inter-regional functional connectivity and how the pattern of these changes is distributed over the entire brain network. We examined edge-wise differences in the whole-brain functional connectivity between the deaf and hearing adults. Based on the existing literature, we expected that the deaf would exhibit altered connectivity between auditory and visual, somatomotor, and attention-related regions. We also hypothesized that functional connectivity changes following early deafness would extend beyond the auditory system. Specifically, we expected to observe a compensatory increase of integration between large-scale brain systems engaged in language and cognitive control, such as salience network, FPN, and DMN.

Second, we were interested in whether the lack of auditory input in early childhood alters the development of modular brain structure. Modular network structure can be quantitatively described by the graph theory measures of modularity and global efficiency, reflecting levels of network segregation and integration (Sporns 2013; van den Heuvel and Hulshoff Pol 2010). These measures have been previously applied to characterize neuroplasticity during development (Chen and Deem 2015) and following brain injury (Nakamura et al. 2009). The lack of auditory input in early childhood may lead to a reshaping of the auditory module, whose nodes are taken over by remaining sensory and higher-order networks. We expected that this process might lead to decreased network modularity and increased integration. Finally, we explored the specific changes in module composition in the deaf compared to hearing controls. As the auditory network, consisting of regions of primary and secondary auditory cortex, interacts with other functional modules during speech comprehension (Alavash et al. 2019), we might expect broad alterations of the whole-brain network modularity in deafness. We examined the difference between the modular structure of group-averaged functional networks and apriori network division into thirteen well-known large-scale systems.

## Method

### Participants

Twenty-five early deaf subjects (15 females; *M*_*age*_ = 27.8 ± 5.2; range 19–37 years) and 29 hearing subjects (16 females; *M*_*age*_ = 27.2 ± 4.7; range 19–37 years) participated in the study. All subjects were right-handed with normal or corrected to normal vision and no neurological or psychiatric diseases. Four deaf subjects and eight hearing subjects were excluded from further analyses due to excessive motion (more than 10% of outlier scans identified by a scrubbing procedure; see Data Processing section) or image acquisition errors. After exclusion, the deaf group consisted of 21 subjects (14 females; *M*_*age*_ = 26.6 ± 4.8; range 19–37 years) and hearing group of 21 subjects (14 females; *M*_*age*_ = 26.6 ± 5.2; range 19–37 years). The groups did not differ in age, sex, or years of education. The etiology of deafness was either genetic (hereditary deafness) or pregnancy-related (maternal disease or drug side effects). The mean hearing loss was 100.2 dB (range 70–120 dB) for the left ear and 101.4 dB (60–120 dB) for the right ear. All subjects had some experience with hearing aids (currently or in the past) but did not rely on them on a daily basis. Based on a self-assessment survey, all subjects were proficient users of Polish Sign Language (*Polski Język Migowy*, PJM, a natural visual-gestural language used by the deaf community in Poland; see Table 1 for details).

**Table 1:** Characteristics of deaf participants**.** Information about language proficiency is based on a self-assessment survey

ID	Sex	Age	Cause of deafness	Hearing loss (left ear/right ear/mean)	Onset of deafness (age in months)	Hearing aid use	Native language (spoken/sign)	Languages primarily used at the time of the experiment	How well subject understand speech with hearing aid	How well subject speaks polish
Sub01	F	30	Hereditary deafness	110/90/100 dB	0	Uses currently	Sign	Sign	Moderate	Well
Sub02	M	27	Maternal disease/ drugs side effect	120/90/105 dB	0	Used in the past	Sign	Sign	Moderately	Poorly
Sub04	M	23	Hereditary deafness	Average: 90–119 dB, profound	0	Uses currently	Sign	Sign & Spoken	Moderately	Well
Sub05	M	27	Hereditary deafness	Average: 90–119 dB, profound	0	Used in the past	Sign	Sign	Poorly	Poorly
Sub06	M	27	Hereditary deafness	Average: 120 dB, severe	0	Used in the past	Spoken	Sign & Spoken	Poorly	Moderately
Sub07	F	27	Hereditary deafness	Average: 90–119 dB, profound	0	Used in the past	Spoken	Sign & Spoken	Poorly	Moderately
Sub08	F	27	Hereditary deafness	Average: 120 dB, severe	0	Used in the past	Sign	Sign	Poorly	Poorly
Sub09	M	27	Hereditary deafness	120/120/120 dB	0	Used in the past	Sign	Sign	Poorly	Well
Sub10	F	32	Hereditary deafness	89/80/85 dB	0	Uses currently	Sign	Sign & Spoken	Moderately	Well
Sub14	F	32	Maternal disease/ drugs side effect	105/115/110 dB	18	Uses currently	Spoken	Sign & Spoken	Moderately	Well
Sub17	F	19	Hereditary deafness	95/100/98 dB	0	Uses currently	Sign	Sign & Spoken	Moderately	Poorly
Sub18	M	27	Hereditary deafness	94/107/101 dB	0	Used in the past	Sign	Sign	Poorly	Poorly
Sub19	F	30	Hereditary deafness	90/90/90 dB	0	Used in the past	Sign	Sign & Spoken	Poorly	Moderately
Sub20	F	25	Hereditary deafness	70/60/65 dB	0	Uses currently	Sign	Sign	Well	Moderately
Sub21	F	37	Maternal disease/ drugs side effect	110/110/110 dB	24	Used in the past	Spoken	Sign	Poorly	Moderately
Sub22	F	20	Hereditary deafness	113/115/114 dB	0	Used in the past	Spoken	Sign & Spoken	Poorly	Poorly
Sub23	M	19	Hereditary deafness	90/110/100 dB	0	Uses currently	Sign	Sign & Spoken	Well	Well
Sub24	F	19	Hereditary deafness	94/103/99 dB	0	Uses currently	Sign	Sign & Spoken	Very well	Well
Sub28	F	30	Hereditary deafness	78/92/85 dB	0	Uses currently	Sign	Sign & Spoken	Poorly	Poorly
Sub29	F	23	Maternal disease/ drugs side effect	102/120/111 dB	1	Uses currently	Spoken	Sign & Spoken	Moderately	Well
Sub31	F	30	Maternal disease/ drugs side effect	100/120/110 dB	1	Uses currently	Spoken	Sign & Spoken	Well	Well

### Data acquisition

Neuroimaging data were collected using Siemens MAGNETOM Tim Trio 3 T scanner with a 32-channel head coil (Erlangen, Germany). Resting-state functional images covering the whole brain were acquired with a gradient-echo planar imaging (EPI) sequence (33 axial slices in interleaved ascending order; repetition time (TR) = 2190 ms; echo time (TE) = 30 ms, flip angle = 90; field of view (FOV) = 192; matrix size = 64 × 64; slice thickness = 3.6 mm; voxel size = 3 × 3 × 3.6 mm). During the 10-min resting-state run, 282 volumes were obtained for each subject. Participants were instructed to relax and focus on the fixation point displayed on the screen. Communication with deaf subjects in the scanner was provided in PJM via webcam video.

High-resolution T1-weighted images were acquired using a magnetization-prepared rapid acquisition gradient echo (MPRAGE) sequence (176 slices; TR = 2530 ms; TE = 3.32; flip angle = 7; FOV = 256; voxel size = 1 × 1 × 1 mm).

### Data processing

Neuroimaging data were preprocessed using the SPM12 toolbox (Wellcome Department of Imaging Neuroscience, Institute of Neurology, London, UK) running on MATLAB 8.3 (R2014a) (Mathworks, Natick, MA). First, resting-state functional images were corrected for acquisition time (slice-timing) and spatially realigned to the mean image using rigid-body registration. Next, outlier scans with a mean signal higher than 3 SD and frame-displacement (FD) higher than 0.5 mm were identified using the Artifact Detection Toolbox (ART; http://www.nitrc.org/projects/artifact_detect/). Only subjects with less than 10% of outlier scans detected were included in the subsequent analysis. There was no significant difference between the deaf and the control group in the mean motion (*t*(39.85) = −0.37; *p* = 0.71) and the number of outlier scans detected (*t*(31.93) = −0.62; *p* = 0.54).

Then, the structural image was coregistered to the first functional volume and functional images, gray matter, white matter (WM), and cerebrospinal fluid were normalized to the MNI space (voxel size: 2 × 2 × 2 mm) using a unified normalization-segmentation algorithm (Ashburner and Friston 2005).

Further data processing for the purpose of functional connectivity analysis was performed using the CONN Functional Connectivity Toolbox v. 17.f [www. nitrc.org/projects/conn/ (Whitfield-Gabrieli and Nieto-Castanon 2012)]. The anatomical component correction (aCompCor) strategy was used to estimate and remove physiological noise (Behzadi et al. 2007). The principal components of the subject-specific WM, CSF, as well as outlier scans detected by the ART procedure and the six rigid-body motion parameters (and their first level temporal derivatives), were removed in covariate regression analysis (Whitfield-Gabrieli and Nieto-Castanon 2012). Finally, the resting-state time series were filtered using a 0.008–0.09 Hz band-pass filter to remove the effect of high-frequency noise and low-frequency drift.

#### Network construction

A brain parcellation containing 264 regions of interests (ROIs) provided by functional neuroimaging data meta-analysis was selected to construct correlation matrices for the purpose of the whole-brain network analysis (Power et al. 2011). This brain parcellation was extensively validated on other datasets and was used to divide the 264 ROIs into 13 large-scale networks (LSNs) (Cole et al. 2014; Power et al. 2011)(. Each ROI was modeled as a 10 mm diameter sphere centered around the coordinates listed by Power et al. (2011). Six ROIs (four cerebellar ROIs and two ROIs covering the inferior temporal gyrus) were excluded from analysis due to incomplete coverage of the brain in some participants. Denoised functional time series were extracted from the remaining ROIs, and Pearson’s correlation coefficients were calculated for each pair of regions. This resulted in one 258 × 258 correlation matrix for each participant. Finally, Fisher’s transformation was used to normalize Pearson’s correlation coefficients into z-scores.

#### Edge-wise comparisons

We aimed to identify inter-regional functional connections for which the connection strength increased or decreased in the deaf group compared to the control group. We used a mass univariate approach implemented in the Network-Based Statistics toolbox (Zalesky et al. 2010) based on independently testing each of the *m* = 33,153 functional connections. We performed a two-tailed t-test with a null hypothesis for each functional connection, assuming no difference in connection strength between deaf and control subjects. Then, we estimated associated *p*-values and corrected with a false discovery rate (FDR), using the bootstrap method with *N*_*per*_ = 10,000 permutations (Genovese et al. 2002).

#### Whole-brain graph measures

We employed graph theory measures of modularity and global efficiency to examine differences in modular network structure between the deaf and hearing controls. First, we created a weighted, undirected graph by proportional thresholding the functional connectivity matrix to retain the top 10–25% functional connections (with a step of 5%). Here we present the results for the remaining 25% of connections. As graph measures depend on network cost (sum of connection strengths) (Rubinov and Sporns 2010), we normalized them – on a subject level – against a set of randomly rewired null networks (Maslov 2002). Specifically, for each functional network, we created 100 null networks with preserved size and degree distribution and random topology. Then, to estimate null distributions of network metrics, we calculated them for the respective set of null networks. Finally, we normalized each functional network metric by dividing it by the mean value of the corresponding null distribution. All graph measures were calculated using the Brain Connectivity Toolbox (Rubinov and Sporns 2010). The modularity of a network quantifies the extent to which it can be divided into modules. Informally, the module is a densely interconnected set of nodes sparsely connected with the rest of the network (Newman 2006). For a weighted network, modularity is calculated by maximizing the modularity quality function:

$$ Q=\frac{1}{v}\sum \limits_{ij}\left({A}_{ij}-\frac{s_i{s}_j}{v}\right){m}_i{m}_j $$

where *A*_*ij*_ is a weighted connection strength between nodes *i* and *j*, *v* is the cost of the network $v=\sum \limits_{ij}{A}_{ij}$, ${s}_i=\sum \limits_j{A}_{ij}$ is the strength of a node, and *m*_*i*_*m*_*j*_ is the Kronecker delta that equals 1 when nodes *i* and *j* belong to the same community and 0 otherwise. To find the community structure by maximizing *Q*, we ran the Louvain algorithm (Blondel et al. 2008; Newman 2006) 100 times per network, and considered the division that yielded the highest modularity value.

Global efficiency *E*_*glo*_ enabled us to quantify a network integration by measuring the length of the shortest paths between pairs of network nodes. In a weighted network, the shortest path can be calculated as the path with the smallest sum of inverse weights since the stronger connections are intuitively associated with more efficient communication. Formally, weighted global efficiency is given by:

$$ {E}_{glo}=\frac{1}{n\left(n-1\right)}\sum \limits_i\sum \limits_{j,j\ne }{\left({d}_{ij}\right)}^{-1} $$

where *d*_*ij*_ is shortest weighted path length between *i* and *j*.

To test whether graph metrics differ between deaf and hearing groups we used the non-parametric Wilcoxon rank sum test (Wilcoxon 1945).

#### Large-scale brain networks

We examined module composition in both groups to explore more specific differences in the modular brain structure between hearing and deaf participants. For each group, we created a single representative network by averaging connection strengths across subjects. We calculated the significance of the connection strength against zero to eliminate insignificant connections in each group-averaged connection matrix. Assessed *p*-values were corrected using the false discovery rate (FDR) method for both groups separately (Genovese et al. 2002). Connections that survived thresholding, i.e. those with p_FDR_ < 0.05, were retained in the group-averaged connection matrix. To establish a representative modular structure, we ran the Louvain algorithm 1000 times for both group-averaged networks and considered runs that produced divisions with the highest modularity value. Finally, we compared the modular structure in the deaf and hearing group with the large-scale network division revealed by resting-state meta-analysis (Cole et al. 2014; Power et al. 2011).

To quantify our findings, we calculated the overlap coefficient between empirically found modules and well-known large-scale brain systems. The overlap coefficient is a measure of similarity between two overlapping sets. Here, as sets we consider subsets of nodes grouped in a large-scale module. Formally, for two sets A and B overlap coefficient is given as

$$ overlap\left(A,B\right)=\frac{\left|A\cap B\right|}{\mathit{\min}\left(\left|A\right|,\left|B\right|\right)} $$

where |·| denotes the number of elements of the set. Note that the overlap coefficient equals one for every pair of sets that *A* ⊆ *B* or *B* ⊆ *A*.

## Results

### Edge-wise functional connectivity differences between the deaf and hearing adults

**Fig. 1:**
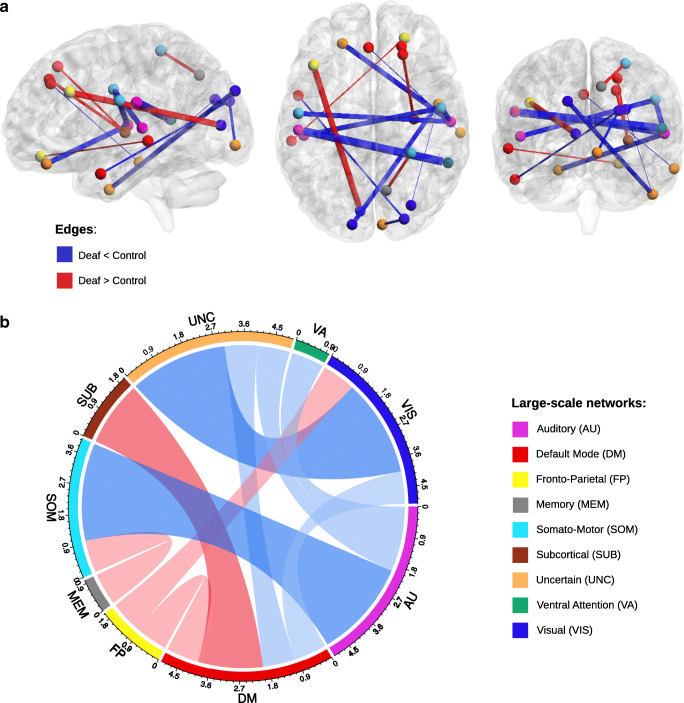
Edge-wise functional network differences visualized (**a**) in brain space and (b) as a chord diagram. (a) Connections that are significantly stronger (red) or weaker (blue) in deaf adults. Edge thickness reflects t-test statistic strength. (b) Chord diagram representing the number of significant edges between different large-scale networks. Red bands represent edges with stronger functional connectivity in the deaf compared to hearing control, while blue bands represent edges with weaker functional connectivity

We compared the strength of all pairwise functional connections (edges) between 258 ROIs in the deaf versus the control group. These comparisons revealed 10 weaker and 5 stronger connections in early deaf adults (Fig. 1**,** FDR corrected *p* < 0.05). Weaker connections in the deaf relative to the controls were found mostly between the auditory and somatomotor networks, as well as between the visual network and regions not assigned to any large-scale networks. Interestingly, stronger connections in the deaf were found between regions beyond the auditory network. These included two enhanced connections between the default mode network (DMN) and the subcortical network. Enhanced connections were also found between the fronto-parietal (FPN) and DMN, between the FPN and visual networks and between the memory and somatomotor networks (see Fig. 1B for edge counts after large-scale network assignment).

### Differences in whole-brain graph measures

**Fig. 2:**
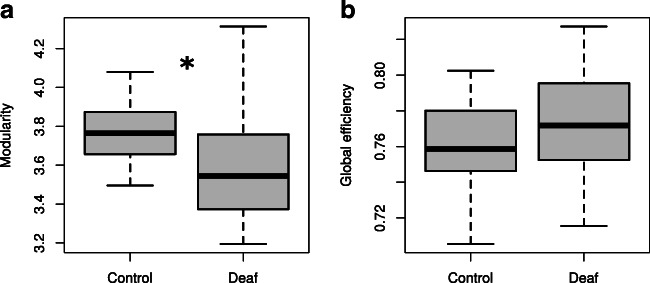
Differences in graph measures of cortical segregation and integration between deaf adults and the control group. (**a**) The difference in network segregation measured as modularity. (**b**) The difference in network integration measured as global efficiency. Boxplots represent topological values calculated for 25% threshold. * *p* < 0.05

Functional brain network topology is believed to support an optimal balance between functional segregation and integration, enabling complex network dynamics (Tononi et al. 1994). These two network features can be captured using two graph theory measures: modularity index for segregation (Newman 2006) and global efficiency for integration (Latora and Marchiori 2001). Here, we tested whether these measures differ between deaf and hearing subjects (Fig. 2). Analysis performed on brain graphs parcellated with 258 functional ROIs revealed significant group differences in network modularity (*z-val* = −2.36; *p* = 0.019, Wilcoxon rank sum test, see Methods). Whole-brain modularity was lower in deaf participants (Q_deaf_ = 3.50; std.(Q_deaf_) = 0.31) than in hearing participants (Q_control_ = 3.65; std.(Q_control_) = 0.14). The variance of the modularity was significantly higher in the deaf than in hearing controls (*F*(20,20) = 4.56, *p* = 0.0013). Lower modularity in the deaf adults was consistently observed for functional networks constructed for all threshold values (*p* < 0.05) (see Methods section for more details on thresholding procedure). This finding suggests that early auditory deprivation may result in weakened modularization of the brain network. The difference in functional network integration measured as global efficiency (*z-val* = 1.26; *p* = 0.21, Wilcoxon rank-sum test) was not significant. These results imply that functional brain networks in early deaf adults are less segregated than those in hearing adults.

### Group-average modular organization

**Fig. 3:**
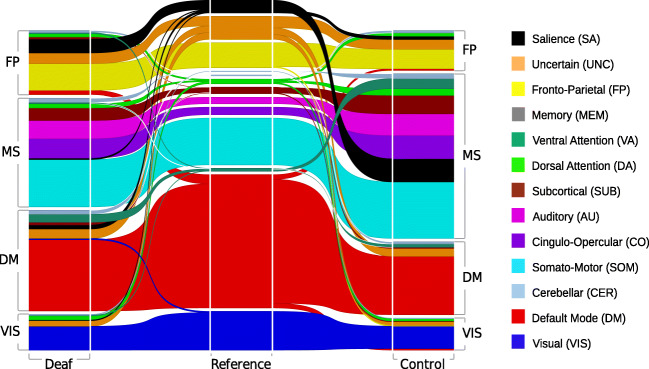
An alluvial diagram representing the segregation of group-averaged networks using a data-driven approach in the deaf (left side of the diagram) and the control group (right side of the diagram). This segregation is then compared against a priori segregation into 13 well-known networks based on meta-analysis studies (Power et al. 2011), shown in the middle column, and described in the right-hand side legend. Note that salience nodes (black) are part of the fronto-parietal (FP) module in the deaf group but fall into the multi-system (MS) module in the control group. Also, the ventral-attention nodes (dark green) are part of the MS module in the control group, but in the deaf group, they are part of the default mode module (DM). The composition of the last visual module (VIS) is highly consistent in both groups

In the analysis that followed, we assessed the modular division of the group-averaged networks using a data-driven approach (see **Methods)** (Blondel et al. 2008). We found that for both groups this approach returned a connectivity structure arranged into four large-scale functional modules (Fig. 3**,** Fig. S1): the fronto-parietal (FP) module, the multi-system (MS) the default mode (DM) module, and the visual module (VIS). In both groups, we then analyzed the overlap of these four modules with 13 well-known large-scale networks (LSNs) that were defined a priori based on meta-analyses (Power et al. 2011) (Fig. 3) by calculating an overlap coefficient between the data-driven modules and all 13 LSNs. In this analysis, an overlap coefficient of 100% means that a given network (for example, the somatomotor network) is completely included in a given module (for example, the multi-system module).

The first module, the FP module, consisted mostly of regions from the fronto-parietal network (overlap in the deaf group (X^fp^_deaf_,X^fp^) = 100%; overlap in the control group (X^fp^_control_,X^fp^) = 96%). This module had a significantly different composition in the deaf as compared to the control group. In the deaf, the salience network contributed significantly more to the FP module than in the group of hearing adults (overlap in the deaf group (X^fp^_deaf_, X^sal^) = 72.2%; overlap in the control group (X^fp^_control_,X^sal^) = 22.2%; Fig. 3, black).

The second module (referred to here as the multi-system module) was the largest and most diverse module (|X^ms^_deaf_| = 78; |X^ms^_deaf_| = 99). In the control group, it was composed of the somatomotor, salience, auditory, cingulo-opercular, ventral-attention, subcortical and cerebellum nodes (overlap >66%). Salience and ventral-attention networks contributed significantly more to the multi-system module in the control group than in the deaf group (overlap(X^ms^_deaf_, X^sal^) = 5.5%; overlap (X^ms^_deaf_, X^va^) = 11.1%). In the deaf group, these networks were associated with other modules, i.e., the salience network with the FP module, and the ventral attentional network with the DM module.

The third module, the DM module, had a very high overlap with the DM (overlap in the deaf group (X^dm^_deaf_, X^dmn^) = 93%, overlap in the control group (X^dm^_control_, X^dmn^) = 93%). It consisted of 75 nodes in the deaf group and 66 nodes in the control group. The DM module was larger in the deaf group mostly as a result of large contribution from the ventral-attention nodes (overlap the deaf group (X^dm^_deaf_, X^va^) = 66.7%) which, as mentioned previously, in the hearing group were associated with the multi-system module.

The last module, the visual module, was the most consistent in both groups (overlap(X^vis^_deaf_, X^vis^_control_) = 88.6%; |X^vis^_deaf_| = 44%; |X^vis^_control_| = 45%). In both groups it was composed primarily from visual network nodes. In agreement with the previous modularity analysis, we also found that the group-averaged functional network was less modular in the deaf group than in the hearing group (Q_deaf-av_ = 0.4571; Q_control-av_ = 0.4748).

## Discussion

In this study, we investigated the whole-brain functional organization differences between early deaf and hearing adults. Using the edge-wise approach, we found that deaf adults exhibited weaker connection strengths, especially between the auditory and the somatomotor networks. Besides changes in the functional connectivity of auditory regions, we also found pronounced connectivity differences between regions located outside of the auditory system. These differences included stronger functional connectivity between the FPN and other large-scale networks (salience, visual, memory, cingulo-opercular and somatomotor, DMN) and between the DMN and the subcortical network in deaf adults. Using graph theoretical measures, we showed that deaf adults had a less segregated (modular) functional network. We also found an altered modular organization of functional networks in deaf subjects. Differences were pronounced for the salience and ventral-attention systems: in the control group, they were part of a multi-system module, but in the deaf, they were coupled with the FP and DM modules. These results suggest that compensatory brain plasticity in sensory loss is a combination of changes in the sensory-deprived brain areas themselves and changes in non-deprived brain areas.

### Reduced functional connectivity between auditory and somatomotor areas

Does the pattern of connectivity differ between early deaf adults and hearing controls? In our edge-wise analyses, we found reduced functional connectivity between auditory and somatomotor areas in the deaf compared to the control group (Fig. 1). Producing and understanding spoken language involves the coordinated engagement of the language network and the speech network (Dick et al. 2014). The speech network includes both subcortical and cortical motor, oropharyngeal muscles, and hearing systems. Weaker connectivity between auditory and somatomotor systems in the deaf may be a result of not using the fast feedback language-speech mechanisms in early development. Recent studies on adult deaf signers reported attenuated structural connectivity in sensory-motor areas involved in the perception and production of speech (Finkl et al. 2020). This suggests that circuits engaged in understanding/production of spoken language may not be established in the deaf due to lack of sensory input and not using spoken language. Future studies relating the level of usage and understanding of spoken language in the deaf are needed to verify if such a relationship exists.

Previous results showed cross-modal plasticity of the auditory cortex of the deaf and its engagement in the processing of tactile stimuli (Auer Jr et al. 2007; Karns et al. 2012; Levänen et al. 1998). Yet, the response of the auditory cortex to tactile stimuli may not necessarily be driven by connections between auditory and somatosensory networks. Meredith et al. (2012) showed that a core auditory cortex of early deaf ferrets is responsive to somatosensory stimulation with no altered cortical or thalamocortical connections. As regional changes of connectivity can not explain observed cross-modal plasticity, the *brainstem theory* of cortical cross-modal reorganization was proposed. The auditory brainstem naturally receives projections from the somatosensory cortices and may mediate the observed cross-modal changes in cortical organization. Further studies should explore the relationship between resting-state connectivity between auditory and somatomotor networks and auditory cortex response to tactile stimulation.

Interestingly, weakened functional connections between the somatomotor and sensory-deprived visual network were also reported in early-blind individuals (Jiang et al. 2015; Liu et al. 2007). Although here we focused on the deaf, such similar findings in the blind may suggest the existence of more general mechanisms of the brain network reorganization in sensory-deprived brains. Liu et al. (2007) interpreted their results of weakened connectivity between sensory-deprived areas and the somatomotor network in terms of the general loss hypothesis (Pascual-Leone et al. 2005). According to this hypothesis, the functional organization of the sensory-deprived brain may be generally disrupted because of the lack of sensory input. However, many studies on early sensory deprivation do not support this notion (Bavelier and Neville 2002; Merabet et al. 2005; Théoret et al. 2004). More studies comparing groups of blind and deaf individuals are necessary to establish if such a general mechanism of brain network plasticity in response to sensory-deprivation exists.

### Increased fronto-parietal and default mode connectivity in deafness

Can we observe group differences in functional connectivity beyond the auditory system? Besides the connectivity decreases outlined above, deaf subjects displayed strengthened interconnections, notably with the FPN and DMN. Edge-wise analysis (Fig. 1) revealed an increased coupling between the FPN and visual areas in the deaf compared to controls. The deaf also displayed higher connectivity between the DMN and FPN, the DMN and subcortical network, and weaker connectivity between the DMN and the visual system (Fig. 1).

The FPN is activated during working memory and attentional tasks (Marek and Dosenbach 2018), as well as language control (Wu et al. 2019). Increased activity of parietal and visual areas was previously shown in the deaf during attentional tasks (Bavelier et al. 2000, 2001; Neville and Lawson 1987b). Cardin et al. (2018) found that the organization of the FPN is shaped by early sensory experience and displays enhanced connectivity in the deaf. The altered functional role of the FPN and enhanced visual–FPN interconnections may constitute the neural basis for the congenitally deaf’s superior performance in both sensory attention (Bavelier et al. 2000) and visuospatial working memory (Ding et al. 2015). While deaf subjects consistently outperform hearing subjects in several other visual tasks (Dewey and Hartley 2015; Scott et al. 2014), this occurs almost exclusively under high attentional load (Heimler et al. 2017). We speculate that enhanced connectivity between the sensory and FPN may provide the neural basis for visual compensation mechanisms, by supporting the higher need for visual attention resources in the deaf. More studies relating connectivity results to behavioral performance in the deaf are needed to fully understand the function of the enhanced coupling between FPN and visual networks.

Both the FPN and visual networks are also engaged in the processing of sign language (Bavelier et al. 2008a, 2008b; Buchsbaum et al. 2005). Thus, we can also speculate that using sign language from early childhood can enhance connectivity between visual and FPN. Studies comparing deaf signers, the deaf using spoken language, and hearing signers are necessary to verify this hypothesis.

The FNP, DMN, and salience networks are crucial for both endogenous and exogenous cognitive control (Sridharan et al. 2008). Here, we found that the DMN was more connected to the FPN and subcortical network in the deaf. The DMN is often referred to as a task-negative network due to its anticorrelation with networks related to attentional processing (Fox et al. 2005). Some studies provided evidence that the DMN is associated with internally directed cognitive processing such as mind-wandering or autobiographical memory (Buckner et al. 2008). Yet, a new wave of research provides evidence for an integrative role of the DMN, which may be crucial for higher cognitive functions (Finc et al. 2017; Vatansever et al. 2015). Previous studies also reported higher task-related functional connectivity between areas of the DMN and medial temporal gyri (Malaia et al. 2014), and higher activity of the DMN in patients with long-term bilateral hearing loss (Xu et al. 2017). The central node of the DMN network - posterior cingulate cortex (PCC) - was also suggested to play a role in language processing (Malaia et al. 2012). In line with this research, stronger connectivity between the DMN and the subcortical and fronto-parietal networks may suggest that the DMN is engaged in network integration that is necessary to compensate for the sensory deficits in the deaf.

### Decreased modularity of functional networks in deafness

Does development without auditory input affect the whole-brain modularity in the adult? Network neuroscience studies revealed that the modularization of the brain network increases during neurodevelopment (Baum et al. 2017). Such modularization promotes efficient information processing within specialized functional modules but also enables information exchange between modules (Sporns and Betzel 2016). Here we found that the modularity of the whole-brain functional network was lower in deaf subjects compared to hearing controls. We did not find group differences in network integration (measured as global efficiency). This finding suggests that early deafness may perturb network modularization, thus disrupting boundaries between functionally specialized systems (Fig. 2). Several studies reported disrupted modular organization associated with healthy aging (Geerligs et al. 2015; Song et al. 2012), childhood-onset schizophrenia (Alexander-Bloch et al. 2010), and autism spectrum disorder (Rudie et al. 2012). Our findings provide the first evidence of an altered modular organization of functional networks in sensory deprived subjects. The variability in network modularity was also higher in the deaf group. Similar variability differences were reported in previous literature (Bavelier et al. 1998; Trumpp and Kiefer 2018). We can speculate that this variability may be driven by varied demographics of the study participants, including the cause of deafness, the level of understanding speech, or using spoken language.

Further studies with a larger sample size are necessary to ascertain the source of higher variance in the deaf’s modular brain organization. These results collectively suggest that sensory deprivation can blur the lines between specialized brain subsystems. At the same time, network integration remains at the same level as in normally developing individuals.

### Altered modular structure of functional network in deafness

How does early deafness affect the modular brain structure? To answer this question, we compared the modular structure of functional networks of early deaf adults and hearing controls. First, we found that the salience network contributed more to the FPN in the deaf group, but not in the control group. Second, we found that the DMN in the deaf included the ventral attention system. In the hearing group, the ventral attention system was coupled with the multi-system module (Fig. 3).

The salience network is responsible for identifying behaviorally relevant stimuli, forwarding them to the executive functions network, and mediating higher-order cognitive processes (Seeley et al. 2007). Recent studies also showed that the salience network might play a crucial role in dynamical switching between the FPN and DMN networks during cognitive control tasks processing (Sridharan et al. 2008). The salience network activity is gradually enhanced with increased working memory load, and this enhancement correlates positively with working memory task performance (Liang et al., 2016). It can therefore be inferred that its strengthened association with the fronto-parietal module reflects compensation effects observed as enhanced attentional and working memory abilities in deafness (Bavelier et al. 2000; Ding et al. 2015).

Previous studies also reported altered structure and function of areas belonging to the salience network in the deaf. When compared with the hearing, deaf subjects recruited the salience network more strongly for short-term verbal memory tasks (Bavelier et al., 2008). They also exhibited stronger functional connectivity between salience and auditory structures when processing a visual working memory task (Ding et al. 2016). In contrast, Le et al. (2018) reported decreased activation of areas belonging to the salience network and the DMN during mental rotation task in deaf signers. Additionally, deaf subjects have increased gray and white matter within the salience network (Allen et al. 2008). This structural reinforcement has been suggested to contribute to sign language processing (Kassubek et al. 2004). Collectively, these results suggest that functional network changes in the salience network could support both working memory and sign language processing in the deaf.

The ventral attention network (VAN) is typically recruited by infrequent or unexpected events that are behaviorally relevant and has been implicated in stimulus-driven, involuntary attentional control (Corbetta and Shulman 2002). Here we also found that the VAN contributed to the DMN module in the deaf. We may hypothesize that closer association of the VAN with the DMN in the deaf corresponds to an easier and faster transition between resting state and the action in response to the unexpected input. Compensatory mechanisms lead deaf people to outperform hearing individuals in certain visual tasks, especially when the location or the exact time of onset of the stimulus is unknown (Bavelier et al. 2006; Corbetta and Shulman 2002), or when the stimulus appears outside the central visual field. The lack of auditory signal is compensated for in the deaf by enhanced peripheral visual attention (Lore and Song 1991; Neville and Lawson 1987a, 1987b; Stevens and Neville 2006). These effects make deaf subjects more distractible by peripheral visual input (Proksch and Bavelier 2002), which may enable them to detect unexpected stimuli more quickly and respond to unpredicted cues in sign language. On a more general level, deaf subjects manifest consistently faster reaction times to visual stimuli across a variety of visual tasks (Pavani and Bottari 2012). Stronger DMN-VAN functional connectivity may also be related to altered retinal structure and larger field of view areas observed in deaf individuals (Codina et al. 2011). Collectively, the enhanced coupling between the DMN and VAN in the deaf may reflect their general higher reactivity to visual stimuli in deafness as well as more specific visual attention capacities. Further studies should explore the possible association between DMN-VAN functional connectivity, visual perception, and superior behavioral performance in the deaf.

## Limitations

Differences between deaf subjects and hearing controls are related to both sensory deprivation and using sign language; large scale effects of both factors can be, to a certain degree distinguished (Bavelier et al. 2001; Cardin et al. 2013). Early deaf individuals recruited in our study were both auditory deprived and sign language users; therefore, we were not able to distinguish between these two factors. Most effects that have been specifically associated with sign language acquisition concern differences in hemispheric laterality. A few studies have reported right-hemispheric activation bias in both deaf and hearing signers, relative to non-signers, for linguistic stimuli (MacSweeney et al. 2004; Newman et al. 2002) and non-linguistic stimuli (Bavelier et al. 2001). Besides the effect of lateralization, deaf signers and deaf non-signers differ in left auditory cortex recruitment when watching sign language. However, these differences were no longer observed when a non-linguistic, purely sensory stimulus was processed (Cardin et al. 2013). This suggests that in the deaf, it is only language-driven plasticity, and not sensory-driven plasticity, that depends specifically on sign language proficiency. Moreover, discrepancies in linguistic plasticity in signers and non-signers are not necessarily related to sign language per se, as different ways of visual communication (lip reading) have been suggested to cause similar cross-modal effects in the auditory cortex (Que et al. 2018). We assume that most of the large scale and bilateral effects revealed in our study are likely related to sensory deprivation rather than proficiency in sign language. Nevertheless, an additional study of early deaf individuals who use only spoken language is needed to tease these effects further apart. Unfortunately, such subjects were not available to us, as a sign language in the Polish deaf population is nearly total.

Moreover, some functional connectivity differences observed between deaf and hearing adults might be related to the bilingual status of some of the study participants that used both sign and spoken language (Wu et al. 2019). Further studies with a larger sample size comparing a group of early deaf signers to a group of early deaf using only spoken language are needed to enable differentiation between these effects (Cardin et al. 2013). Future studies should also include an additional control group of hearing signers, to disentangle the effect of sensory deprivation on sign language acquisition.

## Conclusions

Overall, our results show substantial differences in the functional brain network organization between early deaf and hearing adults. We have shown that deaf adults have reduced coupling between the auditory and the somatomotor cortex. However, we also found many differences in functional connectivity beyond the auditory network, including the fronto-parietal, DMN, and salience networks. These results suggest that brain changes related to sensory deprivation are not limited to the deprived cortices, but manifest in altered connectivity across the entire brain network.

## Electronic supplementary material

ESM 1 (PDF 114 kb)

## Data Availability

The datasets generated and analysed during the current study are available from the corresponding author upon request.
