# Supplementary material for: Early deafness leads to re-shaping of functional connectivity beyond the auditory cortex
Source: Brain Imaging Behav. 2020 Jul 23;15(3):1469–82. doi: 10.1007/s11682-020-00346-y (PMC8286229; doi:10.1007/s11682-020-00346-y)
Supplement: Supplementary file 1 — (PDF 114 kb) [file 11682_2020_346_MOESM1_ESM.pdf]

Early deafness leads to re-shaping of functional connectivity beyond the auditory cortex

Kamil Bonna[1,2*], Karolina Finc[1*], Maria Zimmermann[3], Łukasz Bola[3], Maciej Szul[3], Piotr Mostowski[4], Paweł Rutkowski[4], Włodzisław Duch[1,2], Artur Marchewka[5], Katarzyna Jednoróg[6], Marcin Szwed[3]

[1] Centre for Modern Interdisciplinary Technologies, Nicolaus Copernicus University, Toruń, Poland

[2] Institute of Physics, Faculty of Physics, Astronomy and Informatics, Nicolaus Copernicus University, Grudziądzka 5, 87-100 Torun, Poland

[3]Department of Psychology, Jagiellonian University, Ingardena 6, 30-060, Kraków, Poland

[4] Section for Sign Linguistics, Faculty of Polish Studies, University of Warsaw, ul. Krakowskie Przedmieście 26/28, 00-927 Warsaw, Poland,

[5] Laboratory of Brain Imaging, Neurobiology Center, Nencki Institute of Experimental Biology, Polish Academy of Sciences, Pasteur 3, 02-093 Warsaw, Poland

[6] Laboratory of Psychophysiology, Nencki Institute of Experimental Biology, Polish Academy of Sciences, Pasteur 3, 02-093 Warsaw, Poland

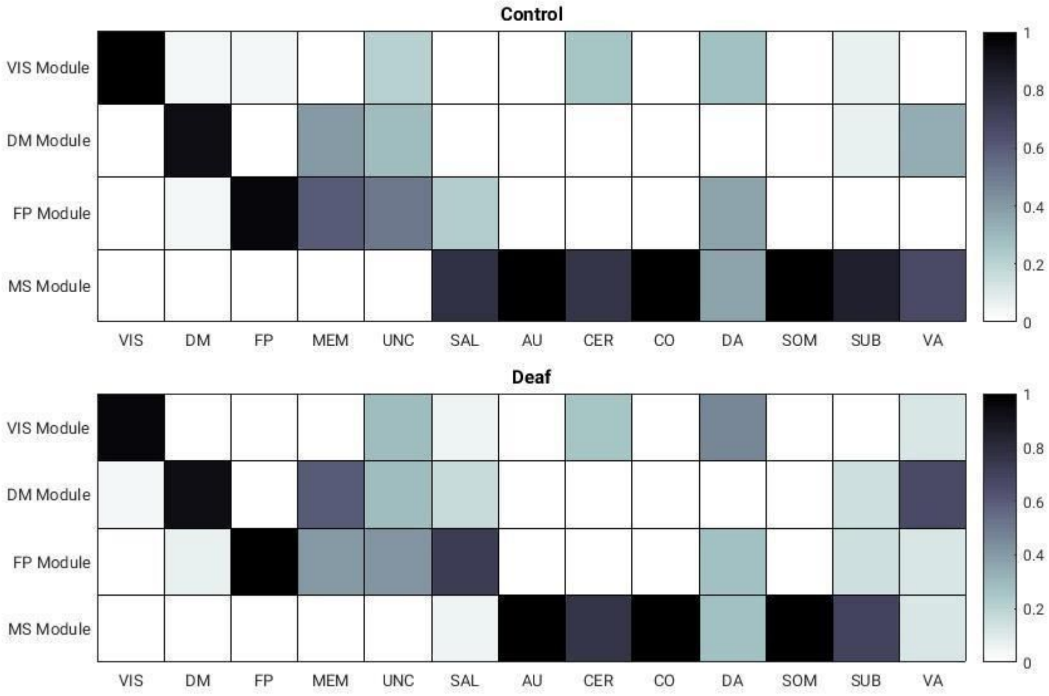

Figure S1. Overlap between data-driven representative network modules in hearing and deaf participants and 13 well-known large scale networks (LSNs) (Power et al., 2011). Vertical axis corresponds to four large-scale functional modules discovered by community detection algorithm (see Results). Horizontal axis corresponds to LSNs from Power et al. (2011). Darker colors reflect higher overlap coefficient between pair of modules.
